# Anti-Fungal Efficacy and Mechanisms of Flavonoids

**DOI:** 10.3390/antibiotics9020045

**Published:** 2020-01-26

**Authors:** Mohammed Saleh Al Aboody, Suresh Mickymaray

**Affiliations:** Department of Biology, College of Science, Al-Zulfi, Majmaah University, Riyadh Region, Majmaah 11952, Saudi Arabia; m.alaboudi@mu.edu.sa

**Keywords:** flavonoids, fungal diseases, mode of action, antifungal activity

## Abstract

The prevalence of fungal infections is growing at an alarming pace and the pathogenesis is still not clearly understood. Recurrence of these fungal diseases is often due to their evolutionary avoidance of antifungal resistance. The development of suitable novel antimicrobial agents for fungal diseases continues to be a major problem in the current clinical field. Hence, it is urgently necessary to develop surrogate agents that are more effective than conventional available drugs. Among the remarkable innovations from earlier investigations on natural-drugs, flavonoids are a group of plant-derived substances capable of promoting many valuable effects on humans. The identification of flavonoids with possible antifungal effects at small concentrations or in synergistic combinations could help to overcome this problem. A combination of flavonoids with available drugs is an excellent approach to reduce the side effects and toxicity. This review focuses on various naturally occurring flavonoids and their antifungal activities, modes of action, and synergetic use in combination with conventional drugs.

## 1. Introduction

Fungal illness often can be fatal, killing more than 1.5 million a year, and such illnesses have an effect on over a billion peoples in a year. Nevertheless, public health authorities have continued to neglect the issue, although the majority of deaths are from fungal infectious diseases. The severe fungal infections often arise because of other health issues, including acquired immunodeficiency syndrome (AIDS), cancer, asthma, diabetes, organ transplantation, and treatment with corticosteroids [1]. Fungal infections have augmented constantly in the current decennium, mainly in immunocompromised hosts or hospitalized individuals with severe underlying infections [2]. Yeasts are large, widespread opportunistic agents in fungal infectious diseases, and various fungal pathogens have been developed in the past decennium [3]. Among the fungal infections, *Candida*, *Aspergillus*, *Pneumocystis*, and *Cryptococcus* are the main threatening agents globally due to the severity and higher incidence of the diseases [4,5]. It is projected worldwide that these fungal species produce, annually, at least 1.4 million fatalities [6]. *Candida* spp. is the most isolated yeast among systemic fungal infections [7,8]. *Candida* is a genus of eukaryotic fungus comprised of 17 species out of 150, which are well-known causative agents of candidiasis in humans [9]. According to the National Network of Health Security, *Candida* spp. are the third most widespread causative agent of blood culture infections (15%) connected to intensive care units, after other common bacterial pathogens [10]. *Candida albicans* is the most ubiquitous species globally (50–70%), which produces more infectious diseases than the total occurrence of infections produced by *C. glabrata*, *C. tropicalis, C. parapsilosis,* and *C. krusei* [7,11]. These yeasts primarily cause superficial and systemic fungal infections that include biofilm-associated infections candidaemia, and fungemia in patients with malignancies [7,12,13]. *Aspergillus* infections are another foremost infection occurring in recipients of hematopoietic stem cell transplants. About 30% of individuals may die from invasive aspergillosis, and the remaining 50% of deaths may occur by candidemia [6]. *Cryptococcus* spp. is another medically noteworthy yeast species, consisting of 40 species; among them, *C. gattii,* and *C. neoformans* are the most clinically applicable [14]. In addition, *C. albidus* and *C. laurentii* are developing pathogens that are participated in various kinds of infectious diseases [15,16,17]. Cryptococcosis is greatly connected with AIDS and meningitis [18]. This infection normally takes place exogenously through breathing or by direct inoculation into the host tissue [3].

The growing resistance of microbes against exiting antifungal drugs is one of the main issues among researchers and clinicians. Pathogenic fungi, viruses, bacteria, and protozoa are more challenging to treat with the existing drugs due to the development of resistance [19,20]. Numerous investigations related to antimicrobial resistance estimated that the mortality rate may go above 10 million by 2050, possibly leading to higher mortality when compared to malignancies and metabolic diseases [21,22,23,24]. The resistance of pathogenic fungi to available antibiotics has developed into a global epidemic. Therapeutic agents for fungal infections are negligible when related to therapeutic agents for bacterial infections [25,26]. In order to heal fungal infections, four categories of antifungal drugs are often offered; viz., polyenes (amphotericin B, nystatin, candicidin, pimaricin, methyl partricin, trichomycin), azoles (fluconazole, itraconazole, ketoconazole, miconazole, clotrimazole, voriconazole, posaconazole, ravuconazole), echinocandins (caspofungins, micafungin, and anidulafungin), and flucytosine (5-fluorocytosine). However, those antifungal agents are only partially effective, and many of them produce several complications to host tissues. Based on a recent therapeutic search, limited antifungal agents have only been structurally and systematically elucidated in the past 30 years [24].

The development of resistance is habitually occurring by antifungal agents that usually bind with cell walls or biosynthetic pathways. For instance, there has been elevated use of fluconazole and amphotericin B, owing to their effectiveness and low toxicity and binding potential toward the membranes of fungal pathogens, consequently stimulating drug resistance [2]. *A. fumigatus* and *C. krusei* are fundamentally resistant to most azole class drugs; viz., fluconazole, itraconazole, voriconazole, and posaconazole. Similarly, *Cryptococcus neoformans* are resistant to fluconazole and echinocandins [6]. Hence, it is an urgent need to investigate novel drugs that have greater anti-fungal activity. The approaches of traditional plant-based medicine or bioactive natural products are great, as such therapeutic medicine can better the prevailing fungal treatments with lesser side effects [24]. 

Medicinal plants with ethnopharmacological uses of crude material or pure compounds have been applied comprehensively for treating and preventing human diseases since time immemorium. These traditional plant approaches have been supported to produce bioactive compounds to recent medicine as therapeutic tools [27,28]. Phytocompounds or bioactive compounds play a significant role in drug discovery by serving as compounds of interest in their natural form or as templates for synthetic changes [29,30]. Numerous studies have demonstrated that natural phytocompounds have potential antifungal activities [31,32,33,34]. The employment of phytochemicals alone or in combination with conventional drugs signifies a greater alternative to conventional therapy. The mixture of those drugs generally needs a smaller quantity of antimicrobials. Hence, this lesser quantity may lead to reducing the toxicity, resulting in great tolerance to the antifungal agents. Based on the available data, even there has been adequate literature concerning antifungal phytocompounds proceeding 2015 [2,35,36,37,38]; only a few studies have reviewed the antifungal flavonoids that were accounted for later in 2015. Hence, this review aimed to focus on the antifungal activities of flavonoids and their modes of action.

An electronic hunt was performed using Google Scholar, PubMed, and Science Direct, and by finding the keywords “Flavonoids” AND “antifungal agents” AND “flavones” or “flavonols” or “flavanones” or “isoflavones” or “flavones” or “flavane” or “anthocyanidins” in “Title/Abstract/Keywords,” with a date cutoff, and checking all available findings (case-control studies, placebo, clinical, in vitro, and in vivo) that examined the relationship between flavonoids and their antifungal effects. Each antifungal mechanism was collected and arranged in an appropriate place for the review.

## 2. Fungal Diseases and Their Complications 

Fungal cells are eukaryotic, and similar to mammalian cells in that they possess nuclei containing DNA, cytoplasm, mitochondria, endoplasmic reticulum, and the Golgi apparatus. However, the fungal cell membrane is made up of ergosterol, which differs from the mammalian cell, which mainly contains cholesterol. The occurrence of ergosterol has been noted as the main drug target of interest in the investigation of antifungal drugs [39]. Fungal cell walls are generally rigid and cover complex polysaccharides called chitin, which are mixtures of β-(1,3)-glucan and β-(1,6)-glucan. The pathogenesis of fungal infections is normally aid by adhesion factors of the cell surface, which initially bind to the host surface. Then, the pathogen secretes membrane discharging hydrolytic enzymes with virulence factors for the invasion that ultimately breaks the host tissues [40]. The occurrence of fungal infections is swiftly growing, which creates significant issues for healthcare specialists. In addition, these infections are rapidly increasing in prevalence with the diseases of cancer, AIDS, and diabetes, and in immunocompromised individuals. They often affect the skin, keratinous tissues, and mucous membranes, which distresses millions of people globally [41]. They ultimately cause devastating effects on the quality of an individual’s life and spread infections to other individuals to become invasiveness. 

Early diagnosis permits rapid antifungal treatment; conversely, it is regularly deferred or unattainable, leading to chronic illness, impaired vision, or death. Since 2013, LIFE-Worldwide (Leading International Fungal Education) portal has initiated and enabled the assessment of the encumbrance of serious fungal infections worldwide since 2013. The annual global accounts have showed the numbers of affected individuals with various fungal infections; viz., skin, hair, nail infections (≈1,000,000,000), recurrent vulvovaginal candidiasis (≈134,000,000), rhinosinusitis (≈12,000,000), fungal asthma (≈10,000,000), allergic bronchopulmonary aspergillosis in bronchial asthma individuals (≈4,800,000), chronic pulmonary aspergillosis (3,000,000), oral candidiasis (≈2,000,000), oesophageal candidiasis (≈1,300,000), invasive candidiasis (≈750,000), *Pneumocystis* causing pneumonia (≈500,000 cases), invasive aspergillosis (≈250,000), cryptococcal meningitis connecting HIV/AIDS (≈223,100), disseminated histoplasmosis (≈100,000), and fungal keratitis (≈1,000,000) [1,18,42,43]. 

*C. albicans* is a typically polymorphic fungus, which is the major cause of invasive candidiasis, a superficial or deep tissue fungal infection. Notably, this invasive candidiasis causes an undesirably high mortality rate worldwide. For instance, *C. albicans* causes the occurrence of oral candidiasis in 80–95% of HIV/AIDS individuals (with a minimum of one episode) [44,45]. Moreover, chemotherapy, certain steroid drugs, treatment with multiple antibiotics, immunosuppressive therapy, antiretroviral therapy, and removable partial dentures may also impact the severity of oral candidiasis [44,45]. As a result, patients may experience weight loss, dysphagia, and disseminated candidiasis that can be life-threatening, with 35–60% mortality rates [8,9,46,47]. There are numerous and different types of candidiasis; viz., mucosal, cutaneous, antibiotic, and systemic candidiasis. *Candida* spp. has known to be causative of severe candidaemia that can show reduced vulnerability to the existing antifungal drugs [8]. *Candida* spp. causing candidaemia is the widespread nosocomial infection connected with an elevated mortality rate (>49%) in immunocompromised patients [9,47]. In about 40% of cases, the patient with candidaemia has sepsis or septic shock. In tropical countries, *Candida tropicalis* (causing candidiasis) has spread intensely on a global scale, and thus, this microorganism has been declared as an emerging pathogenic fungus. There are numerous reports on resistance to azole and other agents in regard to *C. albicans* and *C. tropicalis* [8,47,48,49]. Vulvovaginal candidiasis is another widespread cause of noteworthy morbidity (5–7%) in adult women and affects 70–75% of women at least one time in a lifetime, mostly between 25 and 34 years of age [50,51]. 

In immunocompromised patients, other often-isolated pathogenic organisms include *Aspergillus* spp., which are found with other common pathogenic organisms; viz., *Candida* spp., *Cryptococcus* spp., *Fusarium* spp., *Trichophyton* spp., *Pneumocystis jirovecii, Histoplasma capsulatum,* *Zygomycete,* *Dematiaceous*, and Mucormycetes. These infectious agents often cause endophthalmitis, fungemia, keratitis, onychomycosis, osteomyelitis, peritonitis, pneumonia, septic arthritis, sinusitis, thrombophlebitis, and vulvovaginitis to the host [52]. Colonization of the mucosal surfaces in the lungs by *A. fumigatus*, *A. flavus*, *A. niger,* or *A. terreus* causes allergic bronchopulmonary aspergillosis, which leads to active disseminated infection. The mortality rates may rise from 50% to 90% based on the host’s immune health, site of infection, and treatment regimen [53]. The recipient of a bone marrow transplant has a greater than 95% mortality rate. Other clinical manifestations of aspergillosis are asthma, cutaneous and wound infections, invasive pulmonary aspergillosis, and *Aspergillus* sinusitis. A study on population-based surveillance for cryptococcosis has shown that the higher densities of *Cryptococcus neoformans* in patients with AIDS produce severe forms of meningitis and meningoencephalitis [54]. 

*Malassezia* is a lipophilic fungal genus that encompasses 14 species found in human skin, which are principally involved in various skin diseases; viz., atopic eczema, dandruff, folliculitis, onychomycosis, pityriasis seborrheic dermatitis, sepsis of neonates, and versicolor [55]. Various *Malassezia* species found at higher population densities, up to 10 million, are present on scalps with dandruff; some *Malassezia* species can produce hypo or hyperpigmentation on the trunk and other locations in human [56]. *Penicillium oxalicum* is an anamorph plant pathogen found to cause opportunistic fungal infection in patients with acute myeloid leukemia, chronic obstructive pulmonary disease (COPD), and diabetes [57]. *Pneumocystis jirovecii* pneumonia is developing as a foremost cause of infection in HIV individuals [42]. The global incidence is believed to be greater than 400,000 individuals yearly [58]. The mortality rate of *Pneumocystis jirovecii* pneumonia is about 30% and can be even greater if the diagnosis is overdue [43]. Another significant fungal infection is mycotic keratitis, or keratomycosis, which is also caused by various fungal genuses; viz., *Candida, Aspergillus, Fusarium, Phoma*, *Mucorales*, and *Basidiomycetes.* It is a corneal infection. This infection remains a cause of severe corneal opacification and visual loss, with projected international trouble for approximately 1.2 million people yearly [59].

*Zygomycosis* is another fatal, opportunistic fungal infection, mainly among patients with hematological malignancies, diabetes, and patients treatedwith the drug deferoxamine [52]. A case-control observational study conducted for 27 cases of mucormycosis patients showed that *Rhizopus*, *Mucor*, and *Rhizomucor* spp. caused up to 75% of cases. The infection affects the patient’s immune system, and thus, these infections can be fatal [60]. Infection with *Entomophthora* species is overwhelmingly found with gastrointestinal basidiobolomycosis [61]. Similarly, infection with *Fusarium* species has been described in immunocompetent patients, and causes a broad spectrum of superficial and disseminated infections which may cause 100% mortality rate [62]. The ingestion of *Fusarium*-contaminated food produces hypersensitivity with mycotoxicosis in a healthy individual (Bennett, 2003). *Tinea capitis* is a widespread cutaneous infection of the scalp caused by *Trichophyton* *violaceum, T. tonsurans,* and *Microsporum* spp. that occurs predominantly in children. The burden of this infection is about 21 million school children of 16 countries. It is especially prominent in sub-Saharan Africans [63,64]. 

## 3. Flavonoids 

**Figure 1:**
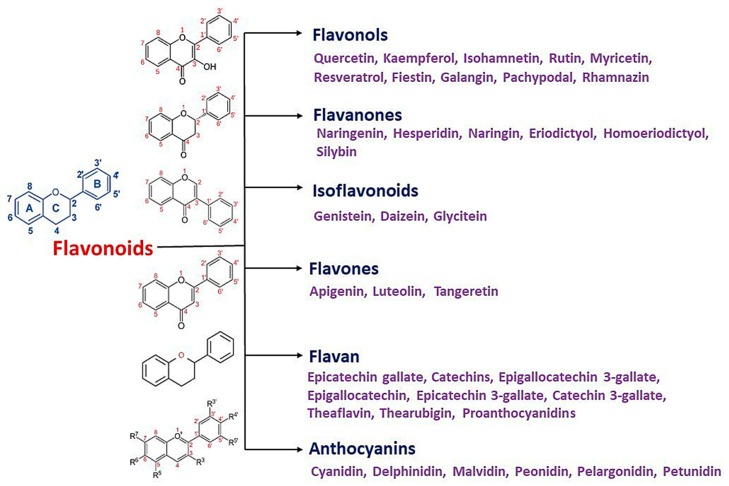
Major classes of Flavonoids.

Flavonoids are secondary metabolites, identified as broad classes of polyphenols that are found largely in plants. These natural compounds greatly exist in foods, including cocoa, onion, apples, bananas, all citrus fruits, grapes, berries, red wine, and sea-buckthorns; and beverages, including, red wine, black tea, green tea, oolong tea, and cider [65,66]. Their broad structure of flavonoids is composed of two phenyl rings coupled together by a 3-carbonated heterocyclic ring (C6-C3-C6) and in total has a 15-C skeleton (Figure 1). According to the changes in the central carbon ring, they can be divided into following subclasses; viz., flavonols, flavanones, isoflavones, flavones, flavan, and anthocyanidins, [65]). Non-cyclization of the C3-portion joints gives rise to chalcones [35], which along with an isoflavonoid unit, pertain to a diverse numbering system [67]. Fl avonoids present along with glycosylated derivatives or acylated with phenolic acids, which have been found in more 6000 in plants. Flavanols and anthocyanidins are generally termed condensed tannins, which are highly complex subclasses and most copious among the flavonoids [35]. 

Several in vivo and clinical investigations have reported that the flavonoids show various pharmacological functions; viz., anti-oxidant [68,69], antidiabetic [70], anti-obesity [71], anti-hyperlipidemic [72], anti-inflammatory [73], antiosteoporotic effect [74], antiallergic and antithrombotic [75], hepatoprotective [76], neuroprotective [77], renoprotective [78], chemopreventive and anticancer [79,80], anti-bacterial, antifungal, and anti-viral activities [81,82,83,84]. Flavonoids can inhibit the in vitro proliferation of various cancer cell lines, and decrease tumor growth in various animal models [85,86,87]. They are recognized as antioxidants and possess free radical quenching properties. Thus, they exert themselves as chelators of divalent cation and free radical scavenger properties that inhibit lipid peroxidation, capillary permeability, and platelet aggregation and fragility [88,89,90]. In addition, flavonoids regulate biological systems through the inhibition of range of enzymes; viz., hydrolase, lipase, α-glucosidase, aldose reductase, cycloxygenase, xanthine oxidase, hyaluronidase, alkaline phosphatase, arylsulphatase, lipoxygenase, Ca+2-ATPase, cAMP phosphodiesterase, and several kinases [91,92,93].

A higher amount or prolonged intake of flavonoids in the diet may cause lesser side effects due to the relatively low bioavailability, lesser intestinal permeability, and higher metabolism rate [94]. Furthermore, flavonoids have only mild toxicity to humans and animals because of their poor absorption coefficient [92,95]. All this information should aid the researcher to explore and investigate the attractive therapeutic indices of flavonoids regarding human wellness. The dietary consumption of flavonoids accounts for 1–2.5 gm/day. The regular consumption of flavonols and flavones has been found to be 23 mg/day, among which, quercetin supplies 16 mg/day. Catechins, quercetin, and isoflavones are measured as the top absorbed compounds; meanwhile tea catechins, condensed tannins, and anthocyanins are the negligibly absorbed compounds [35,93,94].

## 4. Antifungal Activities of Flavonoids

Flavonoids have been found to be effective antifungal agents against a wide range of pathogenic organisms represented in Table 1 [86,96,97,98,99,100,101,102,103,104].

The screening of antifungal flavonoids from plants has been assayed by using broth dilution, spore germination, and agar well or the disk diffusion. Derrone and licoflavone C extracted from *Retama raetam*, which has potent antifungal effects against *Candida* spp. with minimum inhibitory concentrations (MIC) of 7.81 and 15.62 μg/mL, correspondingly [107]. Papyriflavonol A acquired from *Broussonetia papyrifera* that verified as antifungal agents against *C. albicans* with a MIC of 25 μg/mL [119]. A plant-derived flavonoid, Quercetin-3-O-rutinosides had beneficial effects on *C. albicans* and *C. krusei* that exhibited with MICs of 16 and 32 μg/mL respectively [160]. Two distinguished flavonoids, 5,7,4′-trihydroxy-8-methyl-6-(3-methyl-[2-butenyl])-2S- flavanone and 7-hydroxy-3′,4′-methylene dioxy flavan obtained from *Eysenhardtia texana* and *Termanalia bellerica*, which possess potential antifungal properties against *A. flavus* with MICs of 256 and 64 μg/mL respectively [161]. Renowned flavonoids such as quercetin, myricetin, baicalein (from *Scutellaria baicalensis*), gallotannin (from *Syzygium cordatum*), apigenin and kaempferol (from propolis) isolated and reported as potential anti-candidal properties [130,143,155]. In addition, coumarins and lignans have also presented antifungal effects against numerous dermatophyte species [162]. Flavonoids and catechins acquired from Brazilian traditional medicinal plants, *Eugenia dysenterica,* and *Pouteria ramiflora* that have shown potential antifungal activities against *C. tropicalis, C. famata, C. krusei, C. guilliermondii,* and *C. parapsilosis* [163]. 

Various folkloric medicinal plants contain various fractions of flavonoids that show antifungal properties. *Ocotea odorifera contain e*llagitannins, has reported as a fungistatic potential against *C. parapsilosis* [164]. Sanguiin H-6 and lambertianin C and isolated from raspberry (*Rubus idaeus* L.) fruit reported as antifungal effects against *Geotrichum candidum* [165]. *Acacia mearnsii contains* encapsulated tannins that inhibit the effects against *A. niger* and *C. albicans* [166]. *P*ropolis and its high flavonoid content have antifungal activity against dermatophytes and *Candida* spp. Exclusively, propolis contains a flavonol, galangin, which has been demonstrated to have antifungal activities against *Cladosporium sphaerospermum, Penicillium digitatum, A. tamarii, A. flavus, and P. italicum* [167].

Nobiletin, langeritin and hesperidin have extracted from the peels of tangerine oranges and assayed for the activity towards *Deuterophoma tracheiphila* that exhibits promising antifungal activities [168]. The antifungal effects have also been reported in flavonoids extracted from citrus fruits after processing in industries and bergamot peel that averts the growth of *S. cerevisiae* [169]. Quercetin, naringenin is recognized to be potent inhibitors of *C. albicans,* and *S. cerevisiae* [170]. Chlorflavonin is the first chlorine-containing flavonoid type antifungal agent, produced by strains of *A. candidus* [171]. A recognized flavone, baicalein; and flavonol, myricetin have greater inhibitory effects on *Candida* sp., with MICs of 1.9–21 and 3.9–64 μg/mL, correspondingly [97]. 

The antifungal activity of 40 coumarins have studied against *C. albicans*, *A. fumigatus*, and *F. solani*, among them, osthenol and 4-acetetatecoumarin have demonstrated higher antifungal effects [172]. Petroleum ether extracts of *Baccharis darwinii* and *Ferula foetida* contain well-known coumarin, diversinin and 5, 8-dihydroxyumbelliprenin, which have confirmed antifungal activity against *T. rubrum*, *T. interdigitale,* *T. mentagrophytes*, and *M. gypseum* [162]. Phenylpropanoids are natural compounds that classified as coumarins, lignans and phenylpropanoic acid, often investigated due to their anti-*candidal* nature [2]. Scopoletin (coumarin), salicylaldehyde and anisyl alcohol (phenylpropanoic acids) have potential antifungal effects against *C. albicans*, with MICs of 25, 31, and 31 μg/mL correspondingly [173,174]. Similarly, antifungal activities have been described in hesperidin, neohesperidin, naringin which are normally isolated from the citrus fruits. These compounds have strong fungal inhibitory activity against *P. expansum, F. semitectum, A. parasiticus, A. flavus* [175].

Grapes are a rich source of flavonoids, and their pomaces largely help to avert the growth of *Zygosaccharomyces bailii* and *Zygosaccharomyces rouxii* [176]. Chilean grape pomace extract is recognized to have antifungal activity against *Botrytus cinerea* [177,178]. The growth of *C. albicans* could be averted by flavonoid extracts from Brazilian grapes [101]. Similarly, *Eysenhardita texana* has *prenylated flavanones* that have potential antifungal activity against *C. albicans* [112]. Flavanol is generally found in propolis that is also suggested to be used as antifungal agents [179]. Flavonoid extracts of *Sida acuta* Burm f. have shown a varying range of antifungal activity against *C. albicans*. The degree of MIC and Minimum fungicidal concentration of extracts have accounted for 0.078–0.625 mg/mL and 0.078–1.25 mg/mL, correspondingly [180]. Bitencourt et al. [181] demonstrated that the four flavonoids such as quercetin, ellagic acid, galangin, and genistein have shown the most potential antifungal property with MIC of 125, 250, 1000, 1000 µg/mL against *Trichophyton rubrum*, which is common species among the fungal associated dermatophytosis. This team has further reported the antifungal potential of flavonoids that have been recognized as FAS inhibitors which modulate the fatty acid synthesis gene expressions in *T. rubrum*. The crude and butanolic leaf extract of *Terminalia catappa* contain the active components of hydrolyzable tannins (punicalin, punicalagin), gallic acid, and flavonoid C-glycosides that exhibits antifungal activity against *Candida* sp. [182]. Similarly, crude and ethanol leaf extracts of *Carya illinoensis contain* gallic acid, ellagic acid, rutin, catechins and epicatechins that exhibits antifungal activity against different *Candida* strains with MIC range of 6.25–25 mg/mL [183]. 

Gallic acid is established to have potent antifungal property against *Candida* spp., and *T. rubrum*. Gallic acid is isolated from acetone fraction of *Buchenavia tomentosa* that inhibits the proliferation rate and disrupts 48 h-biofilm abruptly in *C. albicans* [184]. Ethyl acetate and butanolic extracts of *Punica granatum contain* ellagic acid, gallagic acid, punicalins, and punicalagins which show antifungal activities against *C. albicans*, *C. neoformans*, and *A. fumigatus* [185]. Curcumin is a renowned flavonoid present in turmeric, which has potential anti-candidal activity against various clinical isolates of *C. albicans* [186] *and* *C. gattii* [187]. Curcumin can decrease the colony width, sprouting, and sporulation of *A. flavus and C. albicans* [188]. Similarly, Curcumin-silver nanoparticles have also exhibited potential anti-candidal activity against *Candida* species acquired from clinical samples of infected HIV individuals with MIC range of 31.2–250 μg/mL [189]. All these findings strongly recommend that flavonoids exhibit a broad spectrum of antifungal activity against *Candida* spp., *Aspergillus* spp., *Geotrichum* spp., *Cladosporium* spp., *Penicillium* spp., *Deuterophoma* spp., *Trichophyton* spp., *Trichophyton* spp., *Dermatophyte* spp., *and Fusarium* spp.

## 5. Mechanism of Actions of Antifungal Flavonoids

**Figure 2:**
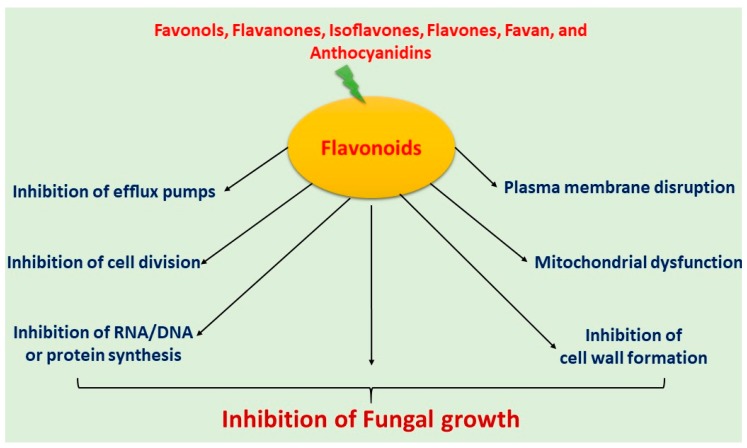
Mechanism of antifungal activity of flavonoids.

Flavonoids have been extensively used for many centuries in the treatment of the range of human diseases. Flavonoids often inhibit fungal growth with various underlying mechanisms, including plasma membrane disruption, the induction of mitochondrial dysfunction, and inhibiting the following: cell wall formation, cell division, RNA and protein synthesis, and the efflux mediated pumping system (Figure 2).

### 5.1. Induced Plasma Membrane Disruption

The ergosterols are a vital component for the manufacturing of cell membranes. Antifungal drugs normally inhibit the ergosterol biosynthesis, and the cell membrane’s integrity is perhaps disrupted, leading to leakage of intracellular components [190,191]. This inadequate formation or disruption of the plasma membrane leads to a lesion or membrane permeability changes [192]. Furthermore, excess production of reactive oxygen species (ROS) also causes severe oxidative stress to the cell, which results in the progressive membrane permeabilization, or injury to nucleic acids and oxidation of fatty acids and amino acids [193,194,195]. ROS often encounter the membrane lipids in *C. albicans* and generate lipid hydroperoxides; this is known as lipid peroxidation [196].

Lipid peroxidation has been demonstrated to disturb the lipid bilayer and alter membrane potentials, resulting in reduced fluidity, increased permeability, and disruption of phospholipids [197]. The relationship between ROS generation and the lipid bilayer leads to the synthesis of malondialdehyde, which is a chief marker of lipid peroxidation. Apigenin has exerted antioxidant and antifungal activity against *C. albicans, C. parapsilosis, Malassezia furfur, T. rubrum,* and *T. beigelii* all with the MIC of 5 µg/mL. Antioxidant potential of the flavonoid inhibits biofilm formation and stimulates membrane disturbances, resulting in the reduction of cell size and leakage of intracellular components [132]. In the previous study, LicoA demonstrated antifungal activities against *T. rubrum* with MIC of 11.52 μM, and the orientation of genes connected to the pathway of ergosterol biosynthesis [124]. In an earlier study, prenylflavanone 8PP obtained from *Dalea elegans*, had potential antifungal activity against *C. albicans, C. glabrata, C. krusei, C. neoformans,* and *T. mentagrophytes* [135]. In this study, prenylflavanone 8PP potentially inhibited the biofilms of sensitive and azole-resistant *C. albicans* at 100 μM through the gathering and elevation of endogenous ROS and reactive nitrogen intermediates [136]. 

Similarly, Baicalein has been isolated from *Scutellaria baicalensis*, which shows inhibitory effects towards *Candida* spp. when used in synergetic mixture with flucanazole at MIC of 64 μg/mL [129,130,131]. Baicalein has induced the apoptosis through alteration in the membrane potentials of mitochondria and elevates intracellular ROS and upstream regulation of redox-related genes [128]. In another study, baicalein presented antifungal activities toward *T. rubrum*, *C. albicans*, *T. mentagrophytes*, and *A. fumigatus* with MICs of 120, 30, 60, and 230 μM respectively [123]. Baicalein has induced concentration-dependent ROS generation, deformation of membrane structure, and efflux of a cotton-like constituents that are alleged to degenerate cytosol in fungal bodies of *T. rubrum, T. mentagrophytes, A. fumigatus*, and *C. albicans* [123]. However, Kang et al. [129] reported controversial outcomes, including that antifungal screening of baicalein in *C. krusei* isolates showed higher alteration in the mitochondrial homeostasis without elevating the intracellular ROS, thereby causing apoptosis [129]. Antifungal activities of fisetin inhibit the growth of *C. neoformans, C. gattii, M. gypseum, T. mentagrophytes, T. rubrum,* and *T. tonsurans* with MIC range of 4–128 µg/mL. In this study, reductions of ergosterol levels and structural alterations were detected in *C. gattii* [118,198]. 

Fatty acid synthase is a significant enzyme essential for endogenous fatty acid synthesis in the membrane of fungi, indicating it as a potential target for novel antifungal drugs [199]. Quercetin has been reported to have individual or synergic antifungal properties with flucanazole, which is recognized as an inhibitor of fatty acid synthase. The inhibitory effects of quercetin and fluconazole were reported as MICs of 125 and 63 μg/mL against *T. rubrum* [106]. Likewise, catechin or epigallocatechin gallate have also shown synergic antifungal effects with the MIC values of 16 and 1 µg/mL respectively. These active flavonoids induce the activation of phosphatidylserine, which inhibits fatty acid synthase. In addition, they stimulate the intracellular accumulation of ROS, structural modifications, apoptosis, mitochondrial depolarization, and fragmentation of DNA in *C. tropicalis* [101]. Isoquercitrin has also shown antifungal activities against *C. albicans, M. furfur, C. parapsilosis, T. rubrum,* and *T. beigelii* with MIC values of 2.5–5.0 μg/mL through inhibition of fatty acid synthase and plasma membrane disruption [200].

### 5.2. Inhibition of Cell Wall Formation

The cell walls of fungi are primarily composed of β-glucans and chitin. The antifungal mechanism has been based on cell wall deformation which is caused by the inhibition of the synthesis of those compounds [39,191]. Glabridin is a chief active isoflavane isolated from *Glycyrrhiza glabra*, and has significant antifungal activities against *C. albicans, C. tropicalis C. neoformans,* and *C. glabratas* with MIC values ranging from 16 to 64 µg/mL. The antifungal process is achieved based on the cell wall deformation which includes the remarkable decreasing of cell size and increasing membrane permeability [96]. Similarly, glabridin treatment enhances the expression of various genes in *C. glabrata* which participate in the fragmentation of DNA (chromatin condensation) resulting in apoptosis [201]. These deformations of the cell wall normally occur due to the presence of the prenylation of glabridin [201]. Antifungal effects of pedalitin (5,6,3′,4′-tetrahydroxy-7-methoxyflavone) have been reported against several strains of *C. albicans* and *Cryptococcus* spp. [202]. An animal model study against disseminated Candidiasis showed epigallocatechin-o-gallate’s synergistic interaction with amphotericin B against *C. albicans* [177]. Infected animals administered with mixed doses of epigallocatechin-o-gallate and amphotericin B exhibited an augmented survival rate compared to animals administered with amphotericin B. The results show that epigallocatechin-o-gallate exclusively inhibits the hyphal formation and ergosterol synthesis in *C. albicans* [177]. The investigations of propidium iodide assay and artificial membrane permeability study specified that pedalitin stimulates the elevation of permeability and physical alarm of the plasma membrane, permitting the diffusion of molecules smaller than about 3.3 nm. These cell wall deformations and the membrane damage are generally promoted by pedalitin, which contributes to malfunctions of the membrane that causes depolarization, K+ leakage, and reduction in membrane fluidity, eventually leading to cell death [167,203]. 

### 5.3. Induced Mitochondrial Dysfunction

Inhibition of the mitochondrial electron transport chain (ETC) leads to diminishing membrane potential. This inhibition generally takes place in the ETC by inhibition of proton pumps, which reduces ATP synthesis, and thus, cell death [39]. Wogonin (5,7-dihydroxy-8-methoxy flavone) showed antifungal activity against *A. fumigates, T. rubrum*, and *T. mentagrophytes* with MICs of 230, 60, and 60 µM respectively. The treatment with wogonin induces accumulation of ROS in mitochondria and causes a decreased membrane potential and reducing ATP synthesis and eventually contraction or cracking of fungal filaments [123]. Baicalein inhibits biofilm formation in a dose-dependent manner from 4 to 32 µg/mL. The results of confocal scanning laser microscopy, flow cytometry, and transmission-electron-microscopy analysis have shown baicalein treatment reduces cell surface hydrophobicity and mRNA expression, and elevates apoptosis that is connected to the failure of mitochondrial membrane potential [204]. Similarly, quercetin, resveratrol, and curcumin modulate mitochondrial functions by inhibiting oxidative phosphorylation through various mitochondrial enzymes, or by changing the generation of ROS in mitochondria and by modulating the activity of transcription factors which control mitochondrial proteins’ expression [98,99]. All these compounds exhibit pro-apoptotic functions, mediated by the ability to discharge of cytochrome c from mitochondria, or indirectly by upregulating pro-apoptotic proteins of Bcl-2 expressions and downregulating anti-apoptotic proteins [205,206]. Honey extract also contains a flavonoid that improves mitochondrial functions and decreases the vacuolization, adjusting the branching process connected with virulence. Honey extract induces alterations in the cell cycle, membrane integrity, functions of mitochondria, and biogenesis [207]. A synergistic study has also investigated the synergy between epigallocatechin gallate and conventional antimycotics agents, such as miconazole, fluconazole, and amphotericin B, against biofilms of *C. albicans, C. glabrata, C. parapsilosis, C. kefyr, C. tropicalis*, and *C. krusei*. Similarly, epigallocatechin gallate has described as an anti-candidal agent, which has been demonstrated through the mechanism of mitochondrial membrane dysfunction [208]. Likewise, *Spondias tuberosa* rich flavonoids elevate the levels of the superoxide anion via the lysosome, causing hyperpolarization in the mitochondrial membrane, so granting anti-*Candida* activity [209].

### 5.4. Inhibition of Cell Division

The inhibition of cell division generally causes inhibition of microtubule polymerization, which inhibits the mitotic spindle formation ([39]. Honey flavonoid extract inhibits the proliferation of *C. albicans* phenotypes, diminishes the infection, and reduce the distressing membrane integrity. This inhibition is measured by using flow cytometry and scanning electron microscopy analyses. Honey flavonoid extract affects the hyphal transition by decreasing the G0/G1 phase and increasing the G2/M phase [207]. Some flavonoids, such as apigenin, α-naphthoflavone, 3′-methoxy-4’-nitroflavone, and 2′-amino-3′-methoxy flavone, have various ligands of the aryl hydrocarbon receptor that inhibit the cell cycle [210]. Studies show that alizarin and chrysazin suppress biofilm formation in *C. albicans*, and effectively inhibit hyphal formation and inhibit the cell cycle [211]. Another study shows that magnolol and honokiol inhibit the growth of *C. albicans* through the Ras1-cAMP-Efg1 pathway. These compounds have potential inhibitory effects on the cell cycle and biofilm-formation-ability of *C. albicans* [212]. *Rubus chingii* is a well-known traditional Chinese medicinal plant that possesses flavonoid-rich compounds, known to have significant antimicrobial and antifungal activities. The crude extract of this plant synergistically interacts with fluconazole to inhibit *C. albicans*. The probable mechanism behind this synergistic interaction could be the cell cycle arrest at S phase in *C. albicans*. The crude extract containing flavonoids reduce the efflux of Cdr1 ABC transporter, which may be the reason for fluconazole resistance [213]. Similarly, daphnegiravone D, a prenylated flavonoid, has cytotoxic effects and significantly inhibits cell division. Systematically, daphnegiravone D arrests the G0/G1 phase and stimulates apoptosis, by reducing the expression of cyclin E1, CDK2, and CDK4, and promote the cleavage of caspase 3 and PARP [214].

### 5.5. Inhibition of Efflux Pumps

Efflux pumps are transporters present in most living cells, including fungi; they have the noteworthy function of removing toxic substances from the fungal body (Kang et al., 2010). This transporter can detoxify a fungal cell through the removal of a drug being accumulated. The high efflux pump’s expression can lead to drug-resistance. Hence, inhibiting the efflux pumps is a crucial aim for reducing drug resistance [215]. A flavone, 7,4′-dimethoxy apigenin, inhibits the growth of *C. albicans* when synergistically combined with miconazole. This combination reduces ergosterol biosynthesis and inhibits drug efflux pumps with IC_50_ of 51.64 µg/mL [133]. Baicalein (5,6,7-trihydroxy flavone) is a flavone, isolated from *Scutellaria baicalens,* that has significant anticandidal activity with a MIC value of 26 mg/mL. This compound is well recognized as a lipooxygenase inhibitor or efflux pump inhibitor when in combination with fluconazole; it decreases the capacity of the cells to efflux out drugs [127]. Similarly, diorcinol D is another natural compound obtained from a lichen endophytic fungus, *Aspergillus versicolor*, that inhibits the efflux pump activity by decreasing the Cdr1 expression in *C. albicans* [216]. Curcumin from rhizomes of *Curcuma longa* is also another natural polyphenolic compound that modulates the efflux pump activity in *Saccharomyces cerevisiae,* and overexpresses the *C. albicans* ATP binding cassette (ABC) multidrug transporters, Candida drug resistance protein 1, and Candida drug resistance protein 2 [217]. Similarly, the quorum-sensing molecule farnesol is drug efflux a modulator that mediates through ABC multidrug transporters and synergizes with fluconazole, ketoconazole, miconazole, and amphotericin B in *C. albicans.* This synergistic interaction of quorum-sensing molecule farnesol with those antifungal drugs leads to ROS generation, which causes early apoptosis [218]. Naturally occurring flavones, such as apigenin, chrysin, baicalein, luteolin, tangeritin, scutellarein, 6-hydroxyflavone, and wogonin inhibit efflux mediated pumps that induce cell death in the fungi [130,139,219]. An isoflavone, sedonan A extracted from *Dalea formosa*, also inhibits efflux pumps in *C. albicans* and *C. glabrata,* and disturbs various intracellular transcription genes with MIC of 15 and 7.6 mg/mL, respectively [109]. Another isoflavone has been identified as dorsmanin isolated from *dorstenia mannii* that inhibits efflux pumps in *C. albicans* with a MIC of 64 mg/ml [157]. 

### 5.6. Inhibition of RNA/DNA and Protein Synthesis

The antifungal agent generally enters into the cell through active transport that reaches into the nucleus, and thus inhibits DNA, RNA, and protein synthesis. The inhibition of protein synthesis is well-recognized as an antifungal target [39]. For instance, 5-flurocytosine inhibits nucleic acid synthesis by the formation of fluorinated pyrimidine metabolites, which can cause a deficit of cytosine deaminase, resulting in the deregulation of the pyrimidine biosynthesis [220]. Similarly, Catechin inhibits *C. albicans* nucleic acid synthesis; analysis by flow cytometry shows that it exhibits the inhibition of FCS-induced hyphal formation; western blotting results also reveal that the treatment with catechin in the *C. albicans* reduces the hypha-specific gene expression in mitogen-activated protein kinase cascade and the cyclic adenosine 3,5-monophosphate pathway. Based on the findings, the team in question highlighted catechin as a potential antifungal candidate in clinical therapy for the management and prevention of candidosis [221]. Similarly, flavonols (myricetin, kaempferol, fisetin, quercetin, 3-hydroxy flavone, and 3,7-dihydroxyflavone), a flavone (luteolin), a flavanone (naringenin), and isoflavones (genistein, biochanin A) inhibit filamentous fungus *Cochliobolus lunatus* through the inhibition of nucleic acid synthesis [222]. Apigenin is a well-known flavone found in a wide variety of plants and herbs that interferes with the translational activity of fungal foot-and-mouth disease driven by the internal ribosome entry site, and was thus identified as a potential drug for foot-and-mouth disease infection [223]. Carvacrol, a chalcone extracted from *Lavandula multifida* L. that inhibits the nucleic acid synthesis and disrupts the cellular cytoplasmic membrane, eventually causes apoptosis in various candida species [159]. Gallic acid extracted from *Paeonia rockii* inhibits the protein synthesis of *C. albicans,* which has been shown to be involved in a decreasing number of hyphal cells and germ tubes with a MIC of 30 mg/mL [154]. Similarly, gallotannin obtained from *Syzygium cordatum* inhibits RNA synthesis and possess antifungal activity against *C. albicans* with an MIC value of 0.195 mg/mL [155].

### 5.7. Synergistic Action between Flavonoids and Antifungals

The combination of natural products with antifungal drugs is recognized as an effective strategy to fight invasive fungal infections and microbial resistance [224]. This combination is often beneficial and effective for both the rate and degree of microbial killing [225]. Generally, each drug has a diverse mechanism of action, and two drugs may play on diverse targets, resulting in multi-targeting. Based on the multi-targeting strategy, the progress of drug resistance can be reduced [226]. Toxicity and intolerance of the drug can also be evaded with the aid of two or more collective drug treatments. Several in vitro studies have shown a reduced inhibitory concentration of natural products with antifungal drugs [227,228,229]. For instance, bioactive compounds help elevate the intracellular concentration of related antifungals by potentiating their action, inhibiting the efflux pumps, and inhibiting the morphogenesis of drug-resistant *C. albicans* [228]. 

Studies have exhibited that Brazilian Red Propolis and *Acca sellowiana* produce in vitro synergistic activity with fluconazole against resistant fungal isolates of *C. parapsilosis* and *C. glabrata* [227]. Propolis offered action on the cell membrane, permitting fluconazole penetration into the cells [227]. The synergistic effect accelerates between the extracts of *Uncaria tomentosa* and fluconazole against *C*andida non-albicans, and quite likely this effect is connected to teamwork events happening outside the cell membrane [228]. For antifungal therapeutic approaches, a combination of antifungals with the host’s immune system is more essential [171,227,229]. This combination may trigger the healing of lesions and control most of the symptoms connected to fungal infections [229]. Hence, the phytotherapy adjuvant is the main healer for fungal infections exclusively for pharmaceutical companies. 

Curcumin, when combined with fluconazole, miconazole, ketoconazole, nystatin, and amphotericin B in vitro, results in synergistic interaction against *C. albicans* [218]. Curcumin elevates the level of ROS and regulation of expression of numerous genes related to fungal oxidative stress, including superoxide dismutase, catalase, and oxydoreductase [217]. Chalcones are naturally occurring flavonoids that have been synthesized by aldol condensation, which possess significant antifungal properties when combined with fluconazole and resistant strains of *C. albicans*. Chalcones are the main inhibitors of the efflux pump, which in combination with fluconazole decrease the ability of cells to efflux out the drugs [230]. Osthole is a natural methylated derivative of coumarin isolated from *Candida fructus,* which has been extensively used for the treatment of supportive dermatitis and vaginitis in China. It is synergistically combined with fluconazole and possesses significant antifungal effects through the generation of ROS [231]. Similarly, eugenol-tosylate, a semi-synthetic analog of eugenol, has a synergistic interaction with fluconazole that exhibits antifungal activity against fluconazole-resistant *C. albicans* which occurs through the inhibiting of ergosterol biosynthesis [232]. Glabridin exhibits a synergistic combination with fluconazole against resistant strains of *C. albicans,* causing cell wall alteration [233]. Likewise, quercetin’s synergistic combination with fluconazole inhibits *C. albicans* biofilm, which is isolated from vulvovaginal candidiasis patients. These drugs combined, have the ability to avert the adhesion of cell-cell communication and disturb the expression of genes accountable for biofilm formation [234]. 

## 6. Conclusions

The elevation of fungal infections is alarming. They lead to high levels of morbidity and mortality globally. Emerging new fungal species and the incidence of elevated drug resistance for fungal diseases continues to rise. The scenario of the existing antifungal agents and their complications is quite critical. There are limitations manifest by antifungal agents: the lower fungistatic ability, high toxicity, and kidney failure. Hence, it is vital to search novel agents as alternative therapies that are potentially active against most fungal diseases. Medicinal plants containing flavonoids are recognized as safe and endowed with numerous biological functions. Various flavonoids have been extracted and investigated in association with their anti-fungal activities and can be promising, efficient, and cost-effective agents for the inhibition of fungal infections. They often inhibit fungal growth in various underlying mechanisms by enhancing the disruption of the plasma membrane and mitochondrial dysfunction; and inhibiting cell wall formation, cell division, protein synthesis, and the efflux-mediated pumping system. These flavonoids are capable and efficient in synergetic combination therapy with conventional drugs, which can be more appropriate and supportive for finding novel drug therapies against fungal pathogens.

## Figures and Tables

**Table 1:** Antifungal activities of flavonoids.

Flavonoids (Compound Name)	Sources	Structure of the Flavonoids	Fungal Strains Inhibited	MIC *	References
Isoflavonoid glycosides (Dalpanitin)	*Dalbergia scandens* Roxb., Corom.	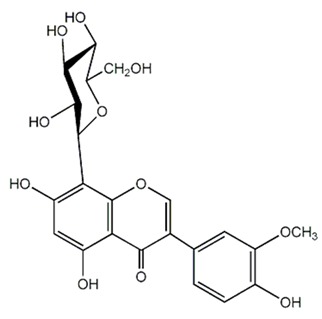	*C. albicans*	780–6250 mg/mL	[104]
**Isoflavones** (Equol)	Soybeans	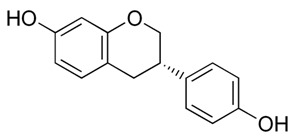	*C. albicans*	516–1032 μg/mL	[105]
**Isoflavones** (Daidzein)	Soybeans	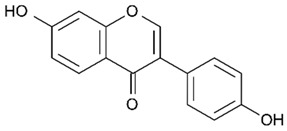	*C. albicans*	516–1032 μg/mL	[105]
Isoflavone (Genistein)	Soybeans	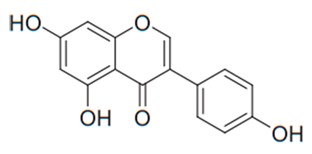	*T. rubrum*	1000 µg/mL	[106]
Isoflavone (Derrone)	*Retama raetam*	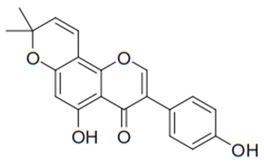	*C. albicans, C. glabrata, C. krusei, C. parapsilosis, C. neoformans*	7.81 µg/mL	[107]
Isoflavanone [(3R)-7-2′-3′-trihydroxy-4′-methoxy-5′-prenylisoflavanon]	*Geoffroea decorticans*	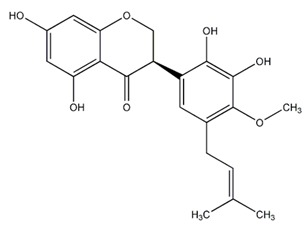	*A. flavus,* *A. parasiticus, A. nomius*	9–18 μg/mL	[108]
Isoflavanone [(3R)-5,7,2′,3′-tetrahydroxy-4′-methoxy-5′-prenylisoflavanone]	*Geoffroea decorticans*	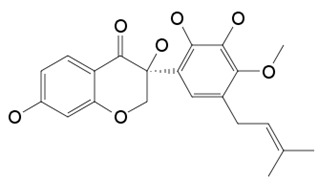	*A. flavus,* *A. parasiticus, A. nomius*	10–21 μg/mL	[108]
Isoflavanone (Sedonan A)	*Dalea formosa*	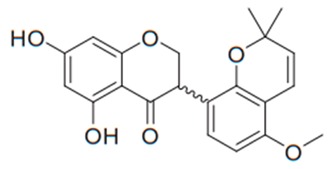	*C. albicans*	7.6–15µg/mL	[109]
Isoflavane (Glabridin)	*Glycyrrhiza glabra*	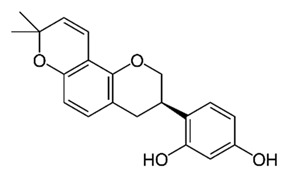	*C. albicans*	6.3–12.5 μg/mL	[110]
Isoflavane (Glabridin)	*Glycyrrhiza glabra*	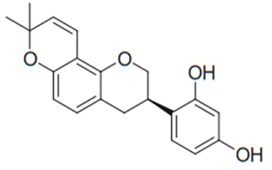	*C. albicans, C. glabrata, C. krusei, C. parapsilosis, C. tropicalis, C. neoformans*	16–64 µg/mL	[96]
Flavonones (Naringenin)	*Kochia scoparia*	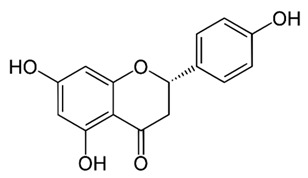	*C. graminicola, T. deformans, A. flavus, H. carbonum, C. zeae-maydis, P. innundatus, S. japonicas, P. herbarum, R. solani.*	3.125 mg/mL	[103]
Flavonones (Hesperetin)	*Baccharis trimera*	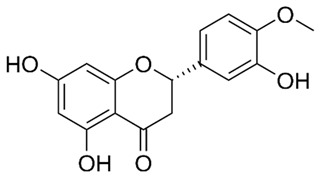	*C. albicans, C. tropicalis, C. parapsilosis, Epicoccum* sp*., C. sphaerospermum, C. neoformans, P. brasiliensis, C. gatti, Pestalotiopsis* sp*., C. lunatus, Nigrospora* sp.	7.8–500 μg/mL	[111]
Flavonones (Eriodictyol)	*Citrus bergamia* Risso	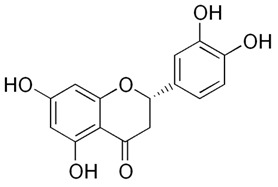	*A. parasiticus, A. flavus, F. semitectum and P. expansum.*	200–800 μg/mL	[112]
Flavonol (Vincetoxicoside B)	*Polygonum paleaceum*	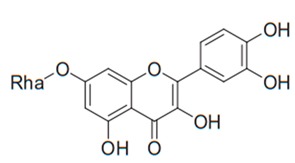	*C. albicans*	64 µg/mL	[100]
Flavonol (Rutin)	Many plants	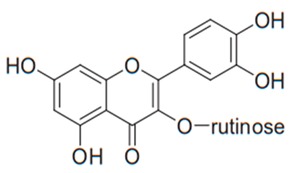	*C. albicans, C. parapsilosis, C. neoformans*	256 µg/mL	[113]
Flavonol (Quercitrin)	*Juglans mollis*	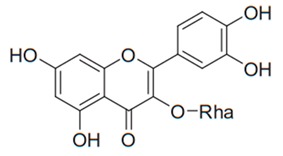	*C. albicans, C. glabrata, C. krusei, C. parapsilosis, C. tropicalis, T. rubrum, T. beigelii*	7.8–256 µg/mL	[97,113]
Flavonol (Quercetin)	Many plants	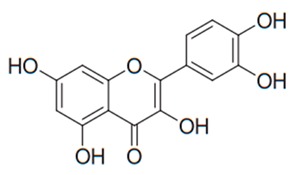	*C. albicans, C. glabrata, C. krusei, C. parapsilosis, C. tropicalis, T. rubrum, T. beigelii*	31.2–125 µg/mL	[97,99,100,101,102,113]
Flavonol (Myricitrin)	*Juglans mollis*	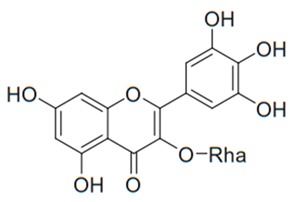	*C. albicans, C. glabrata, C. krusei, C. parapsilosis, C. tropicalis, T. rubrum, T. beigelii*	3.9–83 µg/mL	[97]
Flavonol (Myricetin-3-O-β-glucoside)	*Limonium caspium*	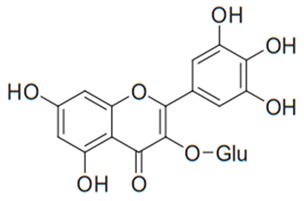	*C. glabrata*	8.53 µg/mL	[114]
Flavonol (Myricetin)	*Myrica rubra*	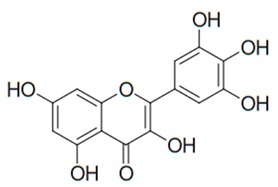	*C. albicans, C. glabrata, C. krusei, C. parapsilosis, C. tropicalis,*	3.9–64 µg/mL	[97,114]
Flavonol (Morin)	*Verbascum glabratum* subsp. bosnense (K. Malý) Murb	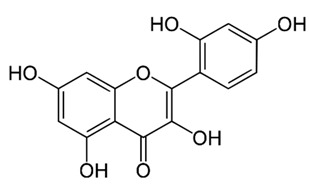	*C. albicans*	600, 1200 μg/mL	[115]
Flavonol (Isoquercitrin)	Aster yomena	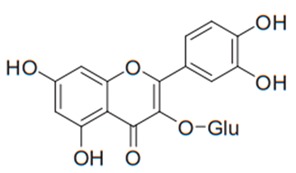	*C. albicans, C. parapsilosis*	2.5–5.0 µg/mL	[116]
Flavonol (Hyperoside)	*Hypericum perforatum*	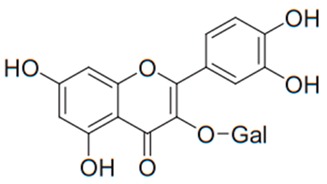	*C. albicans, C. parapsilosis, C. neoformans*	128–256 µg/mL	[113]
Flavonol (Hyperoside)	*Solidago graminifolia* L. Salisb.	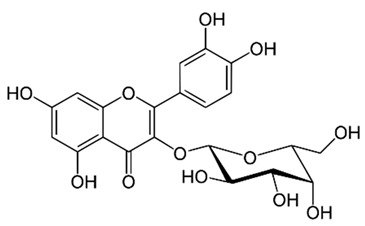	*C. albicans, C. parapsilosis*.	190–6250 μg/mL	[117]
Flavonol (Guaijaverin)	*Myrcia tomentosa*	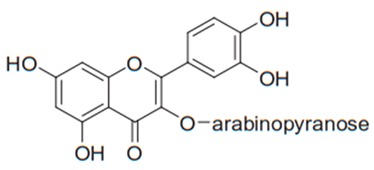	*C. albicans, C. parapsilosis*	2–32 µg/mL	[116]
Flavonol (Galangin)	*Alpinia officinarum*	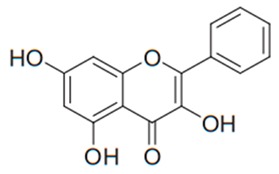	*C. albicans, C. glabrata, C. krusei, C. parapsilosis, C. tropicalis, C. neoformans, C. gattii, T. rubrum*	15.6–1000 µg/mL	[97,106]
Flavonol (Fisetin)	Many plants	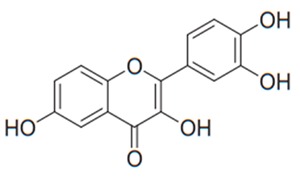	*C. albicans, C. glabrata, C. krusei, C. parapsilosis, C. tropicalis, C. neoformans, C. gattii*	8–128 µg/mL	[97,118]
Flavonol (Avicularin)	*Myrcia tomentosa*	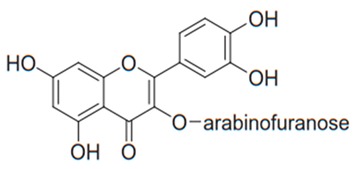	*C. albicans, C. parapsilosis*	2–32 µg/mL	[116]
Flavonol (5-Methylmyricetin)	*Limonium caspium*	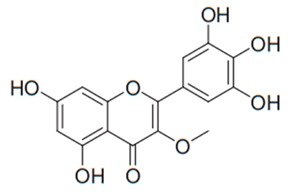	*C. glabrata*	6.79 µg/mL	[114]
Flavonol (Papyriflavonol A)	*Broussonetia papyrifera* (L.) Vent.	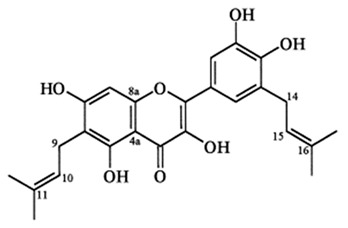	*C. albicans*	25 μg/mL	[119]
Flavonol (Kaempferol)	Many plants	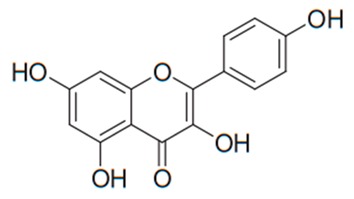	*C. albicans, C. glabrata, C. krusei, C. parapsilosis, C. tropicalis, T. rubrum, T. beigelii*	31.2–512 µg/mL	[97,120,121,122]
Flavonol	Many plants	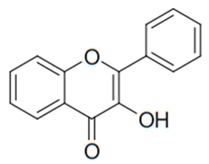	*C. albicans, C. glabrata, C. krusei, C. parapsilosis, C. tropicalis,*	3.9–83 µg/mL	[97,106]
Flavone (Wogonin)	*Scutellaria baicalensis*	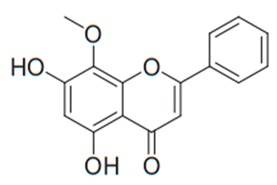	*A. fumigatus, T. rubrum*	60–230 µg/mL	[123]
Flavone (Pedalitin)	*Pterogyne nitens*	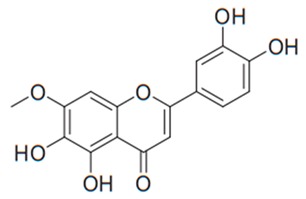	*C. neoformans*	3.9 µg/mL	[123]
Flavone (Luteolin)	*Reseda luteola*	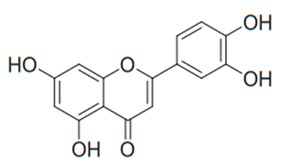	*C. albicans, C. glabrata, C. krusei, C. parapsilosis, C. tropicalis, A. fumigatus, T. rubrum*	3.9–83 µg/mL	[97,124]
Flavone (Luteolin 7-O-β-D-glucuronide)	*Lavandula stoechas Lavandula luisieri* and *Lavandula pedunculata*,	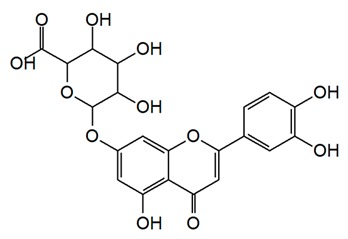	*A. niger*, *C. albicans*, *C. guilliermondii*, *S. cerevisiae*, *C. neoformans*, *R. rubra*, and *T. cutaneum*	7.5–62.5 μg/mL	[125]
Flavone (Luteolin 7-*O*-glucoside)	*Salix babylonica* L.	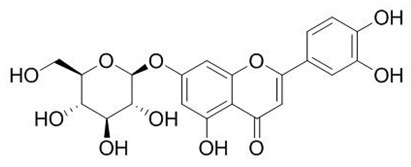	*C. albicans*	1.56–100 mg/mL	[126]
Flavone (Licoflavone C)	*Retama raetam*	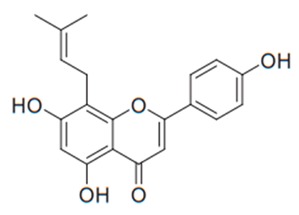	*C. albicans, C. glabrata, C. tropicalis, C. neoformans*	15.62 µg/mL	[107]
Flavone (Baicalin)	*Scutellaria baicalensis*	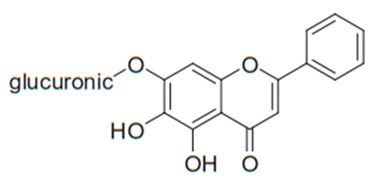	*C. albicans, C. parapsilosis*	250–500 µg/mL	[107]
Flavone (Baicalein)	*Scutellaria baicalensis*	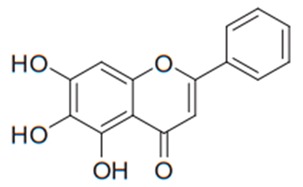	*C. albicans, C. glabrata, C. krusei, C. parapsilosis, C. neoformans, A. fumigatus, T. rubrum*	1.9–64 µg/mL	[123,127,128,129,130,131]
Flavone (Apigenin-7-O-β-glucuronoside)	Oncoba spinosa	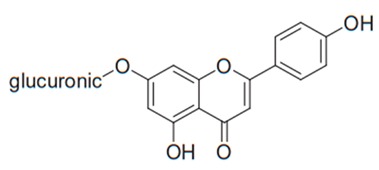	*C. albicans, C. parapsilosis, C. neoformans*	64–256 µg/mL	[113]
Flavone (Apigenin)	Many plants	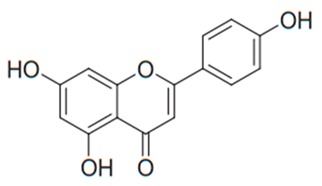	*A. fumigatus, C. parapsilosis, T. rubrum, T. beigelii*	5.0 µg/mL	[132]
Flavone (Apigenin 7-O-β-D-glucoside)	*Lavandula stoechas Lavandula luisieri* and *Lavandula pedunculata*,	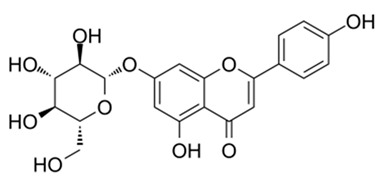	*A. niger*, *C. albicans*, *C. guilliermondii*, *S. cerevisiae*, *C. neoformans*, *R. rubra*, and *T. cutaneum*	7.5–62.5 μg/mL	[125]
Flavone (7,4′-dimethylapigenin)	*Combretum zeyheri*	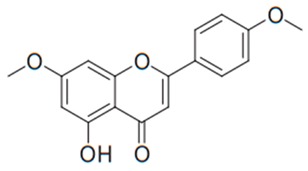	*C. albicans*	10 µg/mL	[133]
Flavone	-	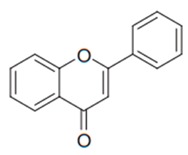	*C. albicans, C. glabrata, C. krusei, C. parapsilosis, C. tropicalis, C. neoformans, A. fumigatus, T. rubrum*	62.5–83 µg/mL	[97]
Flavanone (Pinocembrin)	*Combretum hereroense, Combretum apiculatum, Combretum Collinum*	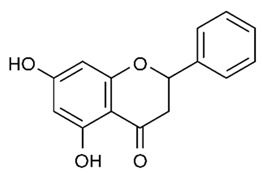	*C. albicans*	6.25 μg/mL	[134]
Flavanone (8PP)	*Dalea elegans*	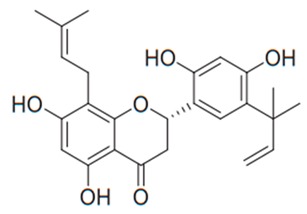	*C. albicans, C. glabrata, C. krusei, C. neoformans*	10–20 µg/mL	[135,136]
Flavanone (Alpinetin)	*Combretum hereroense, Combretum apiculatum, Combretum Collinum*	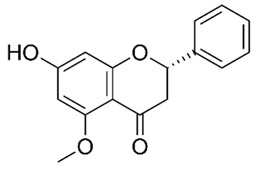	*C. albicans*	25 μg/mL	[134]
Flavanone	*Desmodium caudatum*	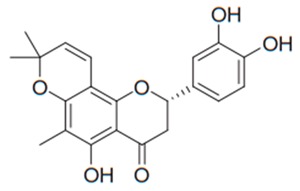	*C. albicans, C. glabrata*	1.95 µg/mL	[137]
Flavan-3-ol (Epicatechin)	*Unonopsis lindmanii* R. E. Fries	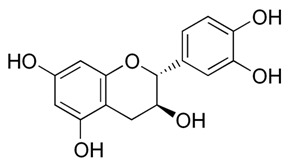	*C. albicans*	25–250 μg/mL	[138]
Flavan (Epigallocatechin gallate)	Tea	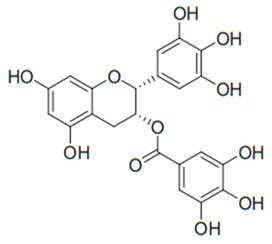	*C. albicans*	15–30 µg/mL	[101]
Flavan (Catechin)	Tea	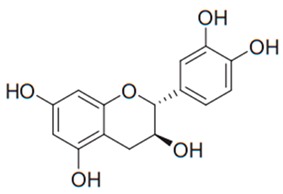	*C. albicans*	15–30 µg/mL	[101]
di-C-glycosylflavones (Schaftoside)	*Solidago graminifolia* L. Salisb.	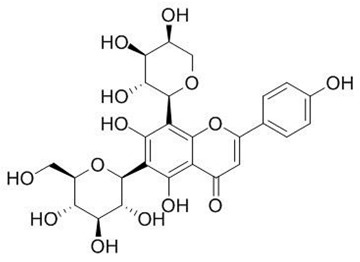	*C. albicans, C. parapsilosis*.	40–3120 μg/mL	[117]
Chalcone (Lico A)	*Glycyrrhiza glabra*	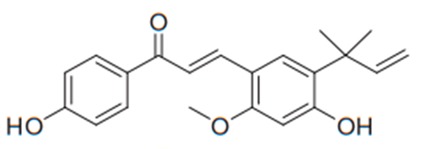	*C. albicans, T. rubrum*	62.5–150 µg/mL	[124,139]
Chalcone (4-hydroxycordoin)	*Lonchocarpus neuroscapha Benth.*	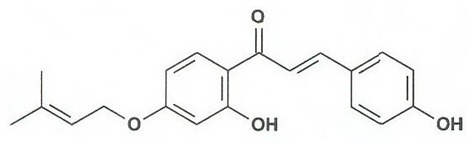	*C. albicans*	50–200 μg/mL	[110]
Chalcone	*Mallotus philippinensis*	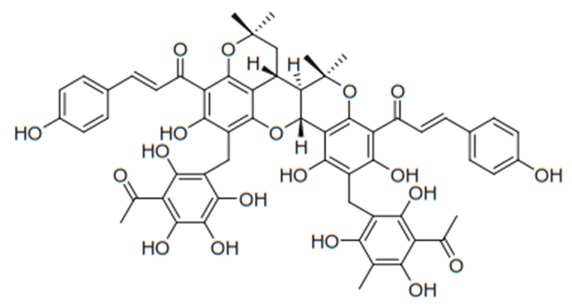	*C. neoformans, A. fumigatus*	4–16 µg/mL	[140]
Chalcone	*Maclura tinctoria* (L.)	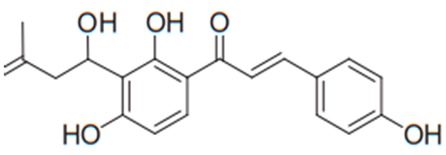	*C. albicans, C. neoformans*	3–15 µg/mL	[141]
Apigenin flavone glucoside (Vitexin)	*Unonopsis lindmanii* R. E. Fries	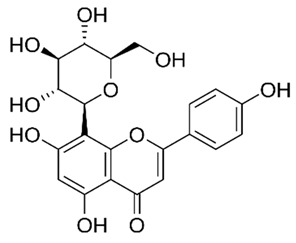	*C. albicans*	25–250 μg/mL	[138]
Anthocyanidins (Peonidin)	*Buchenavia tomentosa* L.	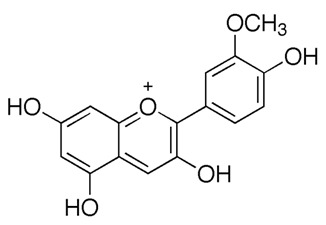	*C. albicans* *, C. tropicalis, C. parapsilosis, C. glabrata, C. krusei and C. dubliniensis.*	200–12500 μg/mL	[142]
Anthocyanidins (Pelargonidin)	*Buchenavia tomentosa* L.	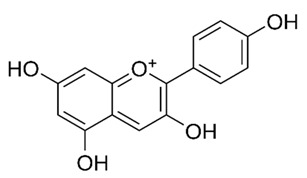	*C. albicans* *, C. tropicalis, C. parapsilosis, C. glabrata, C. krusei and C. dubliniensis.*	200–12500 μg/mL	[142]
Anthocyanidins (Malvidin)	*Buchenavia tomentosa* L.	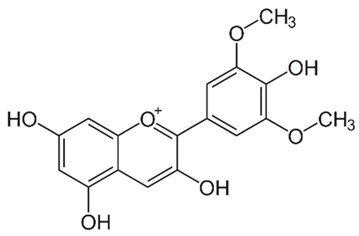	*C. albicans* *, C. tropicalis, C. parapsilosis, C. glabrata, C. krusei and C. dubliniensis.*	200–12,500 μg/mL	[142]
Anthocyanidins (Cyanidin)	*Buchenavia tomentosa* L.	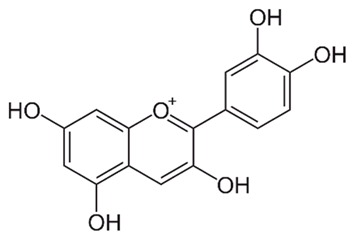	*C. albicans* *, C. tropicalis, C. parapsilosis, C. glabrata, C. krusei and C. dubliniensis.*	200–12,500 μg/mL	[142]
Flavonols (Pinocembrin)	*Propolis*	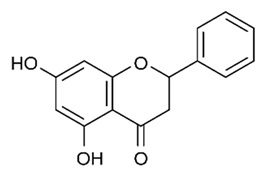	*C. albicans*	197–441 mg/mL	[143]
Flavonols (Talosin A)	*Kitasatos-pora kifunensis*	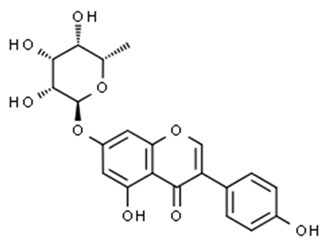	*C. albicans*	15 mg/mL	[144]
Flavonols (Talosin B)	*Kitasatos-pora kifunensis*	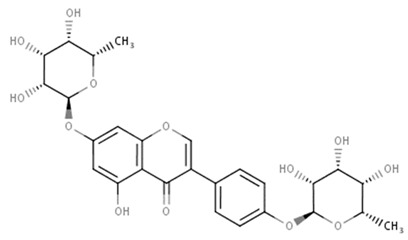	*C. albicans*	7 mg/mL	[145]
Quercetin 3-O-beta-glucoside	*Daucus littoralis Smith*	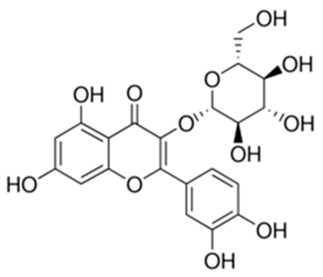	*C. albicans*	7.8 mg/mL	[146]
(R)-roemerine	*Nelumbo nucifera*	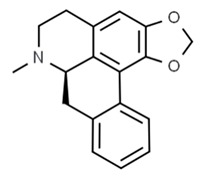	*C. albicans*	16 mg/mL	[147]
Flavones (Robusflavones A)	*Eriosema robustum*	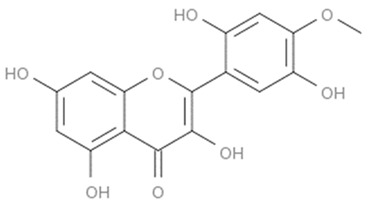	*C. albicans*	160 µg/mL	[148]
Flavones (Conyzoflavone)	*Conyza canadensis*	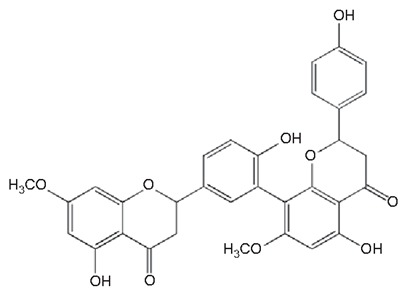	*C. albicans*	10 mg/mL	[149]
Flavones (5,7,3’,4’-tetramethoxyflavone)	*Kaempferia parviflora*	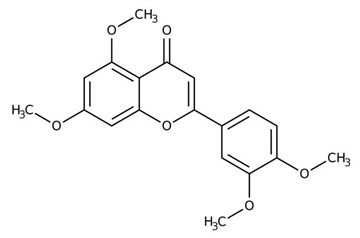	*C. albicans*	39.71 mg/mL	[150]
Flavones (Smiglabrone A)	*Smilax glabra*	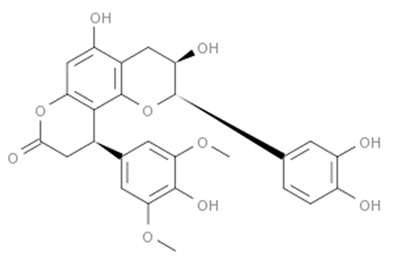	*C. albicans*	146 µg/mL	[151]
Flavones (5,7-dihydroxy-flavone)	*Uvaria scheffleri Diel*	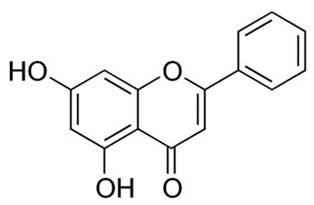	*C. albicans*	31.25 mg/mL	[152]
Flavones (Asterelin A)	*Asterella angusta*	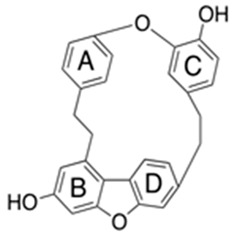	*C. albicans*	16–512 mg/mL	[153]
Flavanols (Gallic acid)	*Paeonia rockii*	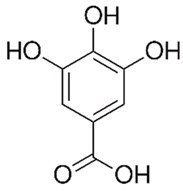	*C. albicans*	30 mg/mL	[154]
Flavanols (Gallotannin)	*Syzygium cordatum*	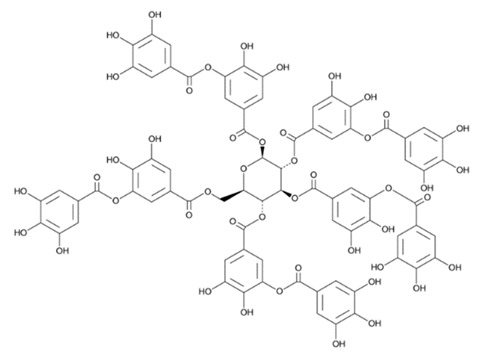	*C. albicans*	195 µg/mL	[155]
Flavanols (1-Galloyl-beta-D-glucopyranosyl-(1!4)-beta-D-galactopyranoside)	*Baseonema acuminatum*	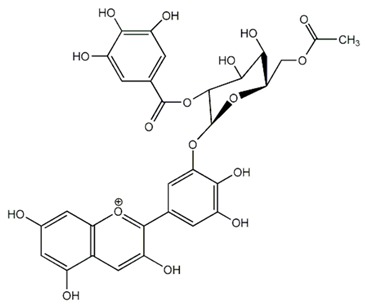	*C. albicans*	12.5 mg/mL	[156]
Isoflavones (Dorsmanin)	*Dorstenia manni*	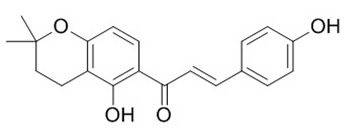	*C. albicans*	64 µg/mL	[157]
Chalcones (2,4-dihydroxy-3-methoxychalcone)	*Zuccagnia punctata*	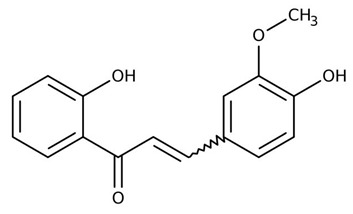	*C. albicans*	400 µg /mL	[158]
Chalcones (2,4-dihydrocychalcone)	*Zuccagnia punctata*	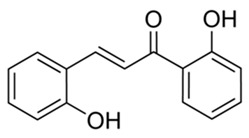	*C. albicans*	400 µg /mL	[158]
Chalcones (Carvacrol)	*Lavandula multifida*	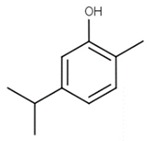	*C. albicans*	160 µg /mL	[159]

* MIC (minimum inhibitory concentration) is the lowest drug concentration at which a given antifungal extract inhibits the visible growth of a tested organism. **MIC absolute value:** the given absolute value of drug concentration inhibits the growth of all tested organisms. **MIC ranges**: the given range of drug concentrations (minimum to maximum) inhibit the growth of the individual to all tested organisms. **Abbreviations:** *A. flavus—Aspergillus flavus; A. fumigatus—Aspergillus fumigatus; A. niger—Aspergillus niger; A. nomius—Aspergillus nomius; A. parasiticus—Aspergillus parasiticus; C. albicans—Candida albicans; C. dubliniensis—Candida dubliniensis; C. gattii—Cryptococcus gattii; C. glabrata—Candida glabrata; C. guillermondii—Candida guillermondii; C. krusei—Candida krusei; C. lunatus—Cochliobolus lunatus; C. neoformans—Cryptococcus neoformans; C. parapsilosis—Candida parapsilosis; C. sphaerospermum—Cladosporium sphaerospermum; C. tropicalis—Candida tropicalis; C. zeaemaydis—Cercospora* zeae-maydis; *F. semitectum—Fusarium semitectum; P. brasiliensis—Paracoccidioides brasiliensis; P. expansum—Penicillium expansum; P. herbarum—Pleospora herbarum; P. innundatus—Protomyces innundatus; R. rubra—Rhodotorula rubra; T. rubrum—Trichophyton rubrum; R. solani—Rhizoctonia solani; S. cerevisiae—Saccharomyces cerevisiae; S. japonicas—Schizosaccharomyces japonicas; T. beigelii—Trichosporon beigelii; T. cutaneum—Trichosporon cutaneum.*
